# Benefits and pitfalls in newborn screening for carnitine uptake deficiency: a 4-year single-center experience

**DOI:** 10.1186/s13023-025-04133-0

**Published:** 2025-11-28

**Authors:** Mariagrazia Turturo, Alessandro Rossi, Ferdinando Barretta, Lucia Albano, Daniela Crisci, Fabiana Vallone, Fabiana Uomo, Simona Fecarotta, Marianna Alagia, Pietro Strisciuglio, Giancarlo Parenti, Giulia Frisso, Margherita Ruoppolo

**Affiliations:** 1 Department of Molecular Medicine and Medical Biotechnology, University of Naples “Federico II”, Naples, Italy; 2 Department of Translational Medicine, Section of Pediatrics, University of Naples “Federico II”, Via Sergio Pansini 5, Naples, 80131 Italy; 3 CEINGE Biotecnologie Avanzate s.c.ar.l, Naples, Italy; 4 Azienda Ospedaliera Universitaria “Federico II”, Naples, Italy

**Keywords:** Inherited metabolic disease, Carnitine transporter, Fatty acid oxidation, Newborn screening, Specificity

## Abstract

**Background:**

Carnitine uptake deficiency (CUD) is an inherited disorder caused by *SLC22A5* gene variants resulting in low plasma and intracellular carnitine concentrations. Although newborn screening (NBS) enables a timely diagnosis of CUD, its efficiency is being debated. The aim of this work was to assess the benefits and limitations of NBS for CUD. A retrospective, observational single-center study was conducted on newborns born between 2017 and 2020 recalled for low free carnitine (C0) values. Biochemical, molecular, dietary and perinatal data were collected. Maternal acylcarnitine profiles and *SLC22A5* genotype were also recorded, if available.

**Results:**

Among 160,015 newborns in the study period, forty-six infants were enrolled in the study: three infants were diagnosed with CUD (incidence 1:53,338 newborns), sixteen infants with a maternal disorder, five infants with one heterozygous *SLC22A5* variant, and nineteen (41%) infants were false positives. Among false positives, additional factors potentially contributing to the observed low C0 values (i.e., gestational age < 37 weeks, low birth weight, C-section, exclusive breastfeeding) were identified. All enrolled infants were asymptomatic. The association of low C0 with concurrent C_tot_ values ≤ 19 µmol/L in dried blood spot appeared to argue against a false positive case.

**Conclusions:**

Collected data showed that NBS for CUD is burdened by a high false positive rate. Besides individuals with CUD, those with maternal disorders and heterozygous individuals who do not require medical care were also identified. Increasing the efficiency of NBS is a compelling need. Possible strategies to minimize false positives include regular revision of diagnostic cutoffs assessment of fractional excretion of carnitine and implementation of new analytical strategies such as the development of second-tier tests.

**Supplementary Information:**

The online version contains supplementary material available at 10.1186/s13023-025-04133-0.

## Background

Carnitine uptake deficiency (CUD, OMIM #212140) is an inherited disorder of the carnitine cycle and transport caused by pathogenic variants in the *SLC22A5* gene, which encodes the organic cation/carnitine transporter 2 (OCTN2) [1]. The ubiquitously expressed OCTN2 is located on the plasma membrane where it enables carnitine uptake by the cells [1]. OCTN2 defect leads to low plasma and intracellular carnitine concentrations, affecting long-chain fatty acid transport into the mitochondria and resulting in impaired fatty acid oxidation (FAO) [2].

CUD usually presents in early childhood. The most common symptoms include (recurrent) hypoketotic hypoglycemic crisis with hyperammonemia and liver dysfunction and elevated CK, which may be precipitated by fasting or infections. Children may also present with hypotonia, skeletal muscle weakness, developmental delay, cardiomyopathy and anemia [3]. Adult individuals may either develop fatigue, decreased stamina and arrhythmia [4] or be asymptomatic [5, 6]. High-dose life-long L-carnitine supplementation is generally effective in preventing major clinical complications in individuals with CUD [7].

Biochemically, CUD is characterized by low serum free (C0) and total (i.e., the sum of free and acylated) (C_tot_) carnitine levels [2]. C0 is detectable by tandem mass spectrometry (MS/MS) on dried blood spots (DBS). Thus, CUD can be diagnosed by newborn screening (NBS) enabling early diagnosis and treatment. Although NBS allows presymptomatic diagnosis in individuals with CUD [8, 9], there is still conflicting evidence regarding its efficiency. Open issues are related to the positive predictive value of NBS and the observation that most of the patients diagnosed with CUD by NBS remain asymptomatic [9–11]. These considerations led to the discontinuation of NBS for CUD in New Zealand [12], warranting additional longitudinal cohort data.

The aim of the current work was to assess the benefits and limitations of NBS for CUD by collecting 4-year single-center biochemical, molecular and clinical data on newborns recalled at NBS and suspected of having CUD.

## Subjects and methods

### Subjects

This was a retrospective, observational single-center study conducted at the Regional NBS reference center Campania, Italy, where NBS samples of all newborns born in the Campania Region were processed. The study was performed in accordance with the Declaration of Helsinki. Collectively, 160,015 subjects were analyzed during the study period.

Subjects were enrolled if they met all the following inclusion criteria: (I) date of birth from 01 January 2017 to 31 December 2020; (II) decreased C0 value (reference value: 11–51 µmol/L) found on more than one NBS DBS sample or decreased C0 value found on a single NBS DBS at a value < 50% lower than the lower reference limit and concurrent low C_tot_ (reference value ≥ 19 µmol/L). Exclusion criteria were: (I) normal C0 value (11–51 micromoles/liter) found on the first and second requested NBS DBS sample and (II) low C0 value on the first DBS sample and normal C0 value found on the second NBS DBS sample.

In our center, for newborns with C0 values < 11 µmol/L, one of the following actions is taken: a request for a new DBS from the birth center, on which a new analysis is performed, or direct refer-ral of the newborn to the clinical reference center for rare metabolic diseases, for clinical evaluation. Based on our experience, gained over the years through the study of newborns diagnosed by molecular investigations, it has been established that for C0 values < 50% of the indicated thresh-old (11 µmol/L) associated with total carnitine (Ctot) values less than or equal to 19 µmol/L, our center directly refers the newborn to the clinical reference center for rare metabolic diseases.In case low C0 value is confirmed on the second DBS sample (and possibly additional DBS requested if the newborn displayed birth weight < 1800 g or underwent parental nutrition or exchange transfusion or intravenous glucose infusion), newborns suspected of having CUD are included in a follow-up program and undergo: (1) routine clinical and biochemical assessment, (2) molecular testing of the *SLC22A5* gene and (3) oral L-carnitine supplementation (100–250 mg/kg/day) initiated upon NBS recall. All caregivers of recalled newborns were instructed to avoid prolonged fasting and to check blood glucose concentrations upon metabolic stress conditions (e.g., intercurrent illness, decreased oral intake) [13]. Furthermore, all mothers of recalled newborns were asked to test their acylcarnitine profile (Supplemental Fig. 1). L-carnitine supplementation discontinuation was proposed to all the subjects in whom ≤ one *SLC22A5* pathogenetic variant was detected.

Subjects enrolled in the present study were divided into four groups: *group 1* (“true positives”) including newborns carrying biallelic pathogenetic *SLC22A5* variants; group 2 (“maternal disorders”) including newborns in whom maternal acylcarnitine suggested a maternal disorder and no *SLC22A5* variant was found; *group 3* (“heterozygous carriers”), including newborns in whom only one *SLC22A5* variant was found, *group 4* (“false positives”), including newborns meeting at least three of the following criteria: (i) normalization of C0 values after L-Carnitine supplementation discontinuation, (ii) no *SLC22A5* pathogenetic variants detected, (iii) normal maternal acylcarnitine profile and (iv) identification of other possible factors affecting C0 values in the newborn (i.e. pregnancy duration, type of delivery, type of milk taken in the first days of life, newborn’s weight). Patients meeting the inclusion criteria who could not be unquestionably assigned to a group were categorized as “unclassified” and included in the cohort calculations. Given the potential influence of birth weight on C0 levels [14, 15], the results from newborns with birth weight below 1800 g are presented separately.

### Methods

All enrolled subjects were clinically and biochemically evaluated upon NBS recall and subsequently every 3–6 months. The duration of the follow up for subjects included in group 1 (“true positives”) is presented in the results (Table 1). Subjects included in groups 2, 3 and 4 were followed on average for 6 months and subsequently discharged home. Collected data were retrieved from patients’ records compiled during routine visits and included: DBS and serum C0 value, serum glucose, ammonia and CK concentrations, liver function tests, blood gases, heart ultrasound, electrocardiogram, weight, height and psychomotor development assessment. Perinatal information of the newborn including duration of pregnancy, type of delivery, birth weight, type of diet (breastfeeding or formula milk) as well as any treatment required in the perinatal period were also collected. Maternal acylcarnitine profile as well as maternal *SLC22A5 *genotype were also recorded, if available.

**Table 1:** Biochemical, molecular and clinical characteristics of patients included in group 1(true positives)

Subjects	C0 first DBS value (reference range 11-51 µmol/l)	Serum C0 follow up first value (reference range 10-44.7 µmol/l)	Serum C0 follow up second value (reference range 10-44.7 µmol/l)	Ctot (normal value ≥19 µmol/l)	Follow up duration (months)	Pregnancy duration (weeks)	Type of Delivery	Birth weight	Type of milk taken in the first days of life	Genotype SLC22A5 cDNA (protein variation) / Family segregation (F/M)	Variant classification			
											refSNP database§ (MAF)*	ClinVar^#^	In silico prediction Score	ACMG^@^
P1	3.54	71	80.6	12.30	6	>37	Natural childbirth	3830 gr	Breast milk	**c.95 A>G (p.Asn32Ser) / F** **c.95 A>G (p.Asn32Ser) / M**	rs72552725 (0.002%)	P	0.53	P
P2	7.7	11.3	40.90	19.80	66	>37	Natural childbirth	3550 gr	Formula milk	**c.34G>A (p.Gly12Ser) / F** **c.95 A>G (p.Asn32Ser) / M**	rs139203363 (0.08%) rs72552725 (0.002%)	CI (2P-2LP-12VUS) P	0.99 0.53	VUS P
P3	5.39	56.8	Not done	15.59	54	>37	Caesarean section	3260 gr	Breast milk	**c.34G>A (p.Gly12Ser) / F** **c.136 C>T (p.Pro46Ser) / M**	rs139203363 (0.08%) rs202088921 (0.08%)	CI (2P-2LP-12VUS) P	0.99 0.87	VUS P

Ctot= Total acylcarnitine *§ NCBI SNPs Database (http://www.ncbi.nlm.nih.gov)* F=Father, M=Mother *MAF: Minor allele frequency (gnomAD) # Clinvar (https://www.ncbi.nlm.nih.gov); In silico prediction score: Aggregated prediction relies on methods such as REVEL and MetaLR (Score 0.9 - 1.0 corresponds with Strong pathogenic, Score 0.8 - 0.9 corresponds with Moderate pathogenic and Score 0.2 - 0.6 corresponds with uncertain significance). @ ACMG criteria (Richards S et al. Genet Med. 2015). VUS= Variant of Uncertain Significance. P= Pathogenetic. LP= Likely Pathogenetic. CI=Conflicting interpretations of pathogenicity

C0 value was assessed on morning samples (≥ 2-hour fasting) through acylcarnitine analysis. Acylcarnitine analysis was performed on DBS sample upon NBS recall and on serum samples during subsequent evaluations by tandem-mass spectrometry (LC/MS-MS) as previously described [16]. In patients who discontinued L-Carnitine supplementation, assessment of serum C0 values was also proposed at least one month after discontinuation. Additional biochemical parameters were evaluated by using routine assays with commercially available kits.

Molecular testing was performed on DNA extracted from EDTA peripheral venous blood samples. All 10 exons and part of the flanking intron regions of *SLC22A5* gene were amplified by polymerase chain reactions and sequenced for mutation analysis, according to standard procedure [17].Variants were reported following the Human Genome Variation Society (HGVS) nomenclature (http://www.HGVS.org/varnomen) and annotated according to NCBI SNPs Database (http://www.ncbi.nlm.nih.gov, accessed July 2025), ClinVar database (https://www.ncbi.nlm.nih.gov/clinvar, accessed July 2025) and American College of Medical Genetics and Genomics (ACMG) guidelines for variant classification [18]. The results of in silico predictions of the missense detected variants were provided as aggregate score, according to Pejaver et al. [19], using the Franklin platform (https://franklin.genoox.com/clinical-db/home).

Statistical analysis was performed using Prism 9.2 software (GraphPad Software, Inc. La Jolla, CA, USA). The comparisons between numerical variables originating from more than 2 independent groups were performed by one-way ANOVA rank test and Dunn’s test. Differences between 2 independent groups were confirmed by the Mann-Whitney test. Correlation analysis was performed by the Spearman’s rank correlation coefficient (ρ). Statistical significance was set at *p* < 0.05.

## Results

Among the 160,015 newborns born in the study period, fifty-four subjects were enrolled in the present study. Of these, 8/54 subjects were excluded from the data analysis due to the lack of at least 2 of the following data: molecular testing, maternal acylcarnitine profile, C0 values after L-carnitine supplementation discontinuation, information on other possible factors affecting C0 values in the newborn(i.e., pregnancy duration, type of delivery, type of milk taken in the first days of life, newborn’s weight). The remaining forty-six subjects were classified as follows: *group 1* (true positives): three subjects, *group 2* (maternal disorders): sixteen subjects, group 3 (heterozygous carriers): five subjects, *group 4* (false positives): nineteen subjects. Three additional subjects (P21, P25, P27) were categorizes as “unclassified” as they could not be unquestionably assigned to group 2 or 3.

**Fig. 1:**
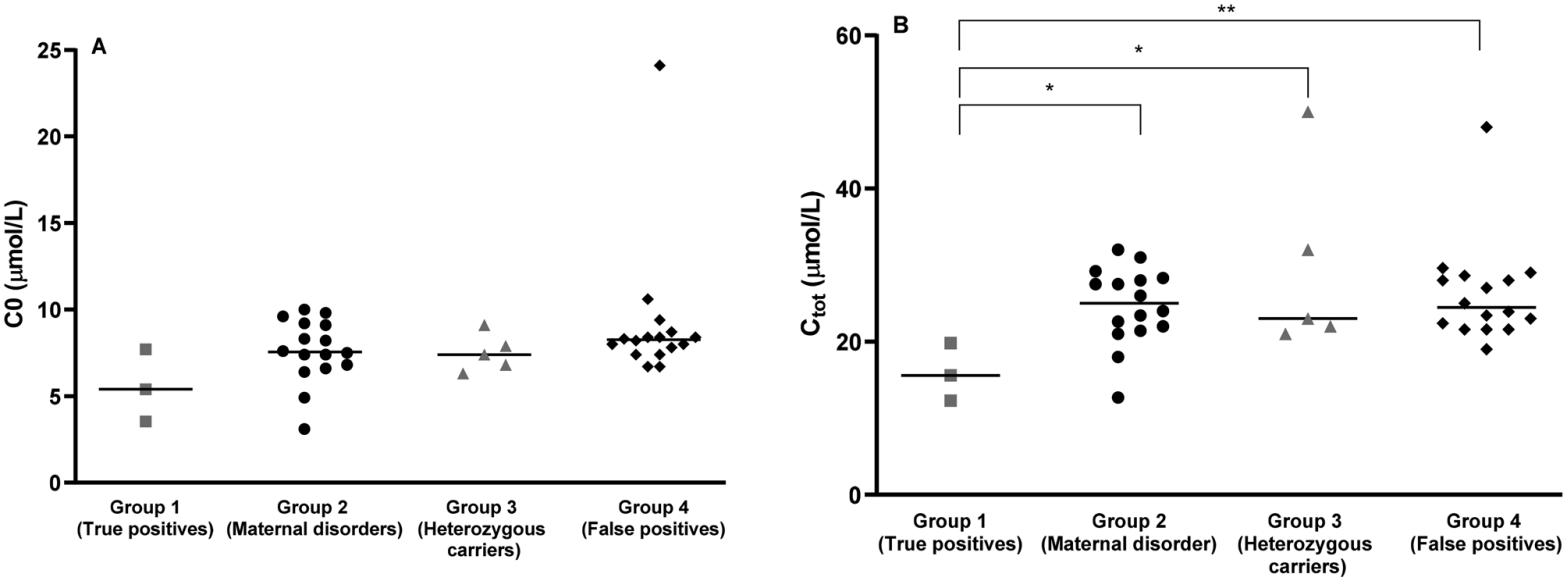
Initial C0 (Panel **A**) and C_tot_ (Panel **B**) DBS value in the study population. Median values are shown. Data from newborns with a birth weight > 1800 g are shown. * *p* < 0.05; ** *p* < 0.01

Three subjects (P1-P3) showed homozygosity or compound heterozygosity for *SLC22A5* variants and were diagnosed with CUD (group 1). Biochemical, molecular, dietary and perinatal characteristics of subjects included in group 1 are shown in Table 1. The incidence of CUD in the study population was 1: 53,338 newborns [18]. The prevalence of CUD among the newborns recalled for low C0 was 6.5% (3/46). In children with CUD, the median C0 value on first DBS was 5.39 µmol/L and the median value of C_tot_ was 15.59 µmol/L (Fig. 1). Among the 3 detected variants, one (i.e. c.34G >A) is classified as a variant of uncertain significance, according to ACMG criteria, and as conflicting variant, according to ClinVar database. This variant was detected in two patients of group 1 in a heterozygous form. All subjects in this group displayed no clinical problems during the follow up. No hypoglycemia, hyperammonemia, metabolic acidosis, elevated transaminases were found. Cardiac evaluation as well as evaluation of the musculoskeletal system and psychomotor development were normal. Patient 3 showed mildly elevated CK values (range 260–340 U/L, reference value < 168) with no associated symptoms.

Biochemical, molecular, dietary and perinatal characteristics of individuals included in group 2 (“maternal disorders”) are shown in Table 2. The prevalence of these newborns among recalled for low C0 was 35% (16/46). The median C0 value on first DBS in group 2 was 7.55 µmol/L and the median C_tot_ value was 25 µmol/L (Fig. 1). Within this group, one mother was molecularly confirmed with type 1 glutaric acidemia (P18). Maternal acylcarnitine profile suggested maternal CUD in other cases (P4-P19). No infants in this group displayed clinical issues during the follow up. Biochemical, molecular, dietary and perinatal characteristics of subjects included in group 3 (“heterozygous carries) are shown in Table 3. The prevalence of heterozygous carriers among newborns recalled for low C0 was 10.9% (5/46). The median C0 value in group 3 was 7.40 µmol/L and the median C_tot_ value was 23 µmol/L (Fig. 1). All subjects in this group had no clinical problems during the follow up. One case of this group was a preterm newborn, with a birth weight < 1800 grams (P26).

**Table 2:** Biochemical, molecular and clinical characteristics of cases relating to maternal disorders (group 2)

Subjects	C0 first DBS value (reference range 11-51 µmol/l)	Serum C0 follow up first value (reference range 10-44.7 µmol/l)	Serum C0 follow up second value (reference range 10-44.7 µmol/l)	Ctot (normal value ≥19 µmol/l)	Pregnancy duration (weeks)	Type of delivery	Birth weight	Type of milk taken in the first days of life	Genotype SLC22A5 cDNA (protein variation)	Variant classification			
										refSNP database§ (MAF)*	ClinVar^#^	In silico prediction Score	ACMG^@^
P4	9.6	73.7	68.1	21.40	>37	Natural childbirth	2920 gr	Formula + breast milk	**WT** **WT**	**_**	**_**	**_**	**_**
P5	4.9	48.2	42.2	18.00	>37	Natural childbirth	3340 gr	Breast milk	**WT** **WT**	**_**	**_**	**_**	**_**
P6	9.8	49.3	Not done	31.00	>37	Natural childbirth	2950 gr	Breast milk	**WT** **WT**	**_**	**_**	**_**	**_**
P7	9.2	20.3	70.3	28.30	>37	Natural childbirth	3300 gr	Breast milk	**WT** **WT**	**_**	**_**	**_**	**_**
P8	8.2	43	Not done	28.00	>37	Natural childbirth	3550 gr	Breast milk	**WT** **WT**	**_**	**_**	**_**	**_**
P9	6.8	62.3	Not done	27.50	>37	Cesarean section	3350 gr	Breast milk	**WT** **WT**	**_**	**_**	**_**	**_**
P10	7.4	138	Not done	24.00	>37	Natural childbirth	3670 gr	Breast milk	**WT** **WT**	**_**	**_**	**_**	**_**
P 11	7.5	67	35.2	21.00	>37	Natural childbirth	3460 gr	Breast milk	**WT** **WT**	**_**	**_**	**_**	**_**
P12	6.4	66.4	Not done	22.60	>37	Natural childbirth	3600 gr	Breast milk	**WT** **WT**	**_**	**_**	**_**	**_**
P13	10.0	17.9	22.4	29.22	<37	Cesarean section	1910 gr	Formula milk	**Not done**	**_**	**_**	**_**	**_**
P14	7.6	Not done	Not done	23.40	>37	Cesarean section	3390 gr	Formula + breast milk	**Not done**	_	_	_	_
P15	9.1	34.4	Not done	27.50	>37	Cesarean section	3270 gr	Formula milk	**Not done**	_	_	_	_
P16	6.6	34	38.4	32.00	>37	Natural childbirth	3550 gr	Formula milk	**WT** **WT**	_	_	_	_
P17	8.3	74.7	23	26.00	>37	Caesarean section	3150 gr	Formula + breastmilk	**c.-149G>A** **WT**	rs57262206 (0.3%)	P	N.A.	P
P18	3.1	54.2	Not done	12.70	>37	Cesarean section	2920 gr	Formula + breast milk	**WT** **WT**	_	_	_	_
P19	7.4	34.9	17	22.00	>37	Caesarean section	3730 gr	Formula milk	**c.95 A>G (p.Asn32Ser)** **WT**	rs72552725 (0.002%)	P	0.53	P

Ctot= Total acylcarnitine §NCBI SNPs Database (http://www.ncbi.nlm.nih.gov) *MAF: Minor allele frequency (gnomAD) # Clinvar (https://www.ncbi.nlm.nih.gov); In silico prediction score: Aggregated prediction relies on methods such as REVEL and MetaLR (Score 0.2 - 0.6 corresponds with uncertain significance). @ ACMG criteria (Richards S et al. Genet Med. 2015). P= Pathogenetic. N.A.= Not Available

**Table 3:** Biochemical, molecular and clinical characteristics of heterozygous carriers (group 3)

Subjects	C0 first DBS value (reference range 11-51 µmol/l)	Serum C0 follow up first value (reference range 10-44.7 µmol/l)	Serum C0 follow up second value (reference range 10-44.7 µmol/l)	Ctot (normal value ≥19 µmol/l)	Pregnancy duration (weeks)	Type of delivery	Birth weight	Type of milk taken in the first days of life	Genotype SLC22A5 cDNA (protein variation)	Variant Classification			
										refSNP database§ (MAF)*	ClinVar^#^	In silico prediction Score	ACMG^@^
P20	7.4	66	Not done	21.00	>37	Natural childbirth	2800 gr	Breast milk	**c.95 A>G (p.Asn32Ser)** **WT**	rs72552725 (0.002%)	P	0.53	P
P22	6.3	12	31.8	23.00	>37	Natural childbirth	3320 gr	Breast milk	**c.131 C>T (p.Ala44Val)** **WT**	rs199689597 (0.0009%)	CI (5LP-2VUS)	0.84	P
P23	6.8	88.9	27.7	22.00	>37	Caesarean section	3200 gr	Breast milk	**c.1345T>G (p.Tyr449Asp)** **WT**	rs11568514 (0.02%)	CI (4P-2LP-6VUS)	0.86	P
P24	9.1	39	31.2	32.00	>37	Natural childbirth	3910 gr	Breast milk	**c.332 A>G (p.Glu111Gly)** **WT**	NR	NR	0.7	VUS
P26	7.9	17.7	40.4	50.00	<37	Cesarean section	1060 gr	Breast milk	**Not done**	_	_	_	_

Preterm newborns with a birth weight<1800 g are displayed in bold (P26)

Ctot= Total acylcarnitine § NCBI SNPs Database (http://www.ncbi.nlm.nih.gov) *MAF: Minor allele frequency (gnomAD) # Clinvar (https://www.ncbi.nlm.nih.gov); In silico prediction score: Aggregated prediction relies on methods such as REVEL and MetaLR (Score 0.8 - 0.9 corresponds with Moderate pathogenic, Score 0.7 - 0.8 corresponds with Supporting pathogenic and Score 0.2 - 0.6 corresponds with uncertain significance). @ ACMG criteria (Richards S et al. Genet Med. 2015). VUS= Variant of Uncertain Significance. P= Pathogenetic. LP= Likely Pathogenetic. CI=Conflicting interpretations of pathogenicity

Biochemical, molecular, dietary and perinatal characteristics of individuals included in group 4 are shown in Table 4. The prevalence of false positive newborns in the study period was 0.012% (19/160,015). The prevalence of false positive newborns among those recalled for low C0 was 41.3% (19/46). The median C0 value on the first DBS in this group was 8.2 µmol/L and the median C_tot_ value was 28 µmol/L (Fig. 1). Three newborns in this group displayed a birth weight < 1800 g (P44, P45, P46). All infants in this group had no clinical problems during the follow up.

**Table 4:** Biochemical, molecular and clinical characteristics of false positives (group 4)

Subjects	C0 first DBS value (reference range 11-51 µmol/l)	Serum C0 follow up first value (reference range 10-44.7 µmol/l)	Serum C0 follow up second value (reference range 10-44.7 µmol/l)	Ctot (normal value ≥19 µmol/l)	Pregnancy duration (weeks)	Type of delivery	Birth weight	Type of milk taken in the first days of life	SLC22A5 genotype
**P28**	**9.4**	**33.6**	**77.5**	**23.40**	**>37**	**Caesarean section**	**3680 gr**	**Breast milk**	**wt** **wt**
**P29**	**8.4**	**31.2**	**33.7**	**29.60**	**>37**	**Natural childbirth**	**1980 gr**	**Breast milk**	**wt** **wt**
**P30**	**7.4**	**Not done**	**Not done**	**23.90**	**>37**	**Caesarean section**	**3670 gr**	**Formula**	**wt** **wt**
**P31**	24.1 *	**38.0**	**38.4**	**48.00**	**>37**	**Caesarean section**	**3180 gr**	**Breast milk**	**wt** **wt**
**P32**	**8.0**	**33.3**	**Not done**	**28.00**	**>37**	**Natural childbirth**	**3970 gr**	**Breast milk**	**wt** **wt**
**P33**	**8.2**	**42.9**	**84.1**	**23.00**	**>37**	**Natural childbirth**	**2940 gr**	**Breast milk**	**wt** **wt**
**P34**	**8.7**	**32.6**	**Not done**	**25.00**	**>37**	**Natural childbirth**	**3480 gr**	**Breast milk**	**wt** **wt**
**P35**	**8.3**	**38.1**	**Not done**	**29.00**	**>37**	**Caesarean section**	**3480 gr**	**Breast milk**	**Not done**
**P36**	**6.7**	**31.6**	**44.0**	**21.60**	**>37**	**Caesarean section**	**3900 gr**	**Breast milk**	**Not done**
**P37**	**8.4**	**47.7**	**Not done**	**27.00**	**>37**	**Natural childbirth**	**2840 gr**	**Formula milk**	**wt** **wt**
**P38**	**7.8**	**20.5**	**34.2**	**21.60**	**>37**	**Caesarean section**	**3320 gr**	**Formula + breast milk**	**wt** **wt**
**P39**	**8.4**	**57.4**	**53.5**	**22.40**	**>37**	**Caesarean section**	**3390 gr**	**Formula + breast milk**	**wt** **wt**
**P40**	**7.4**	**37.2**	**Not done**	**19.00**	**>37**	**Caesarean section**	**3360 gr**	**Formula milk**	**wt** **wt**
**P41**	**6.7**	**50.5**	**27.3**	**21.60**	**>37**	**Natural childbirth**	**3525 gr**	**Breast milk**	**wt** **wt**
**P42**	**1.66**	**54.1**	**33.1**	**28.00**	**>37**	**Natural childbirth**	**2830 gr**	**Breastmilk**	**wt** **wt**
**P43**	**8.0**	**76.7**	**31.3**	**28.60**	**>37**	**Natural childbirth**	**3000 gr**	**Breast milk**	**wt** **wt**
***P44***	22.3*	***53***	***49.20***	***44.20***	***>37***	***Cesarean section***	***800 gr***	***Formula***	***wt*** ***wt***
***P45***	34.7*	***36.6***	***93.20***	***63.00***	***<37***	***Natural childbirth***	***920 gr***	***Formula + breast milk***	***wt*** ***wt***
***P46***	32*	***9.9***	***24.50***	***67.00***	***<37***	***Caesarean section***	***1220 gr***	***Formula milk***	***wt*** ***wt***

Preterm newborns with a birth weight<1800 g are displayed in bold (P44,P45,P46)

*C0 first DBS value was normal, it decreased in subsequent NBS DBS requested because these newborns required glucose infusion and entered a special protocol

**Fig. 2:**
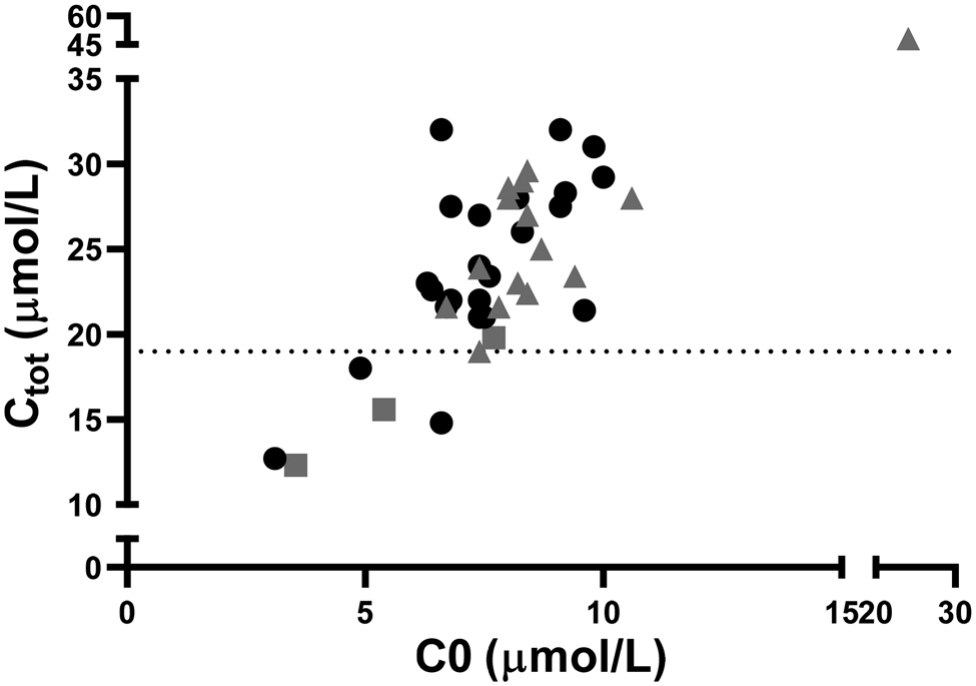
Distribution of C0 and C_tot_ DBS values in the study population. Dotted line corresponds to C_tot_= 19 µmol/L. Data from newborns with a birth weight > 1800 g are shown. True positives, gray squares; Maternal disorders and heterozygous carriers, black circles; False positives, gray triangles

In Fig. 1 initial C0 and C_tot_ DBS values found in the four study groups are presented. The lowest C0 DBS values were detected in one newborn whose mother was subsequently diagnosed with glutaric acidemia type 1 (P18) and in one newborn who carried the homozygous c.95 A > G (p.Asn32Ser) variant in the *SLC22A5* gene (P1). Median initial C0 DBS values gradually increased proceeding from group 1 (true positives) to group 4 (false positives). However, no statistically significant difference was observed among the three groups (*p* = 0.06) (Fig. 1A). Conversely, C_tot_ values significantly differed among the three groups (*p* = 0.03). Specifically, C_tot_ was significantly lower in group 1 compared to group 2 (*p* = 0.01),group 3 (*p* = 0.03) and group 4 (*p* = 0.004) (Fig. 1b). Although a statistically significant correlation was detected between C0 and C_tot_ values in the study population (ρ = 0.64, *p* < 0.0001), no clear group clusterization was found. However, C_tot_ values ≤ 19µmol/L in newborns with low C0 appeared to argue against a false positive case (Fig. 2). When applying this C_tot_ threshold, the sensitivity and specificity for detecting true CUD cases were 100% and 94.7%, respectively, supporting its utility in improving diagnostic accuracy. ROC curve analysis using C_tot_ values yielded an AUC of 0.98.

## Discussion

As a result of the inclusion of CUD in several NBS programs worldwide, the number of patients promptly diagnosed and treated for CUD as well as the phenotype variability have increased over the past years [9–11, 20–24]. However, the efficiency of the current NBS strategy remains to be established. In this study biochemical, clinical and molecular single-center data on newborns recalled for low C0 at NBS over a 4-year interval were collected to evaluate the benefits and limitations of NBS for CUD.

In the present study, molecularly confirmed cases of CUD were 6.5% of the enrolled individuals, with an estimated incidence of CUD of 1:53,338 newborns and a positive predictive value of the NBS for CUD of 0.136 (13.6%). The incidence of CUD is in line with the one reported in Italy [25]. Two patients carried the variant of uncertain significance c.34G >A. This variant has a conflicting classification of pathogenicity: functional study showed that it produces a 50% reduction in carnitine transport compared to control [26, 27]. However, the function of a single missense variant assessed in vitro must be considered cautiously in the disease context. Previous study by Crefcoeur on patient-derived fibroblasts carrying the c.34G >A variant in heterozygosity with another pathogenic variant revealed a residual activity of 26%; these patients were classified as CUD and carnitine supplementation was required [28]. The variant achieved an in silico prediction score of 0.99, exceeding the values estimated for the other variants reported in this study (Table 1), which strongly supported its pathogenic role. Indeed, this variant was found in three newborns who died suddenly [29, 30] and in (mild) symptomatic CUD patients [31, 32]. Furthermore, C0 DBS reassessment tested 8.5 µmol/l and 9 µmol/l (ref 11–51) in our two patients, respectively and their mothers showed no carnitine abnormalities. Hence, they were classified as CUD cases even in the absence of a definitive test, which would have required the measurement of OCTN2 transporter activity in fibroblasts obtained through an invasive skin biopsy. Similarly, carnitine fractional excretion was not assessed, being influenced by supplementation therapy. L-Carnitine supplementation discontinuation was deemed unethical by the managing physicians. In conclusion, even in the absence of a definitive test, we believe that the integrated evaluation of all available (bioinformatic, biochemical, and clinical) information supports the attribution of pathogenicity to the c.34G >A variant, although most likely associated with a mild form of the disease. Further studies on patients carrying the c.34G >A variant in homozygosity are warranted. As only 3 patients with CUD were identified, no genotype-phenotype correlation could be explored in the present study. Indeed, the genotype-phenotype correlation for CUD is not clear yet with likely multiple factors playing a role, as shown in countries with a higher prevalence of CUD cases [33, 34]. Although adverse effects of L-carnitine supplementation were not systematically recorded in this study, no significant side effects were reported during regular follow-up visits.

Infants in whom a maternal disorder (i.e. CUD or GA type 1) was found represented a significant percentage of newborns in the present study (sixteen newborns, 34.8% of newborns recalled). These newborns displayed low C0 levels at birth, which subsequently normalized, even after L-carnitine supplementation discontinuation. All mothers showed an acylcarnitine profile suggesting either acquired/primary carnitine deficiency (in P4-P17, P19) or glutaric aciduria type 1 (in P18) [35, 36]. These data highlight the importance of evaluating the maternal acylcarnitine profile for an adequate interpretation of the DBS result in newborns recalled for low C0.

In the study cohort, 17.4% of newborns recalled for low C0 carried a single variant in the *SLC22A5* gene and were classified as heterozygous carriers. All infants were formally discharged home. Whether heterozygous carriers enrolled in this study will develop metabolic decompensation later in life remains to be ascertained [37]. Hence, families of these children received recommendations on what to do in case of metabolic decompensation and to contact the Metabolic Clinical Center [13]. Five out of eight mothers in this group displayed a normal acylcarnitine profile. Data on acylcarnitine profile were lacking for mothers of 3 children (P21, P25, P27). Despite a single *SLC22A5* variant was found in these children, concurrent primary or acquired maternal carnitine deficiency could not be ruled out in these cases. For three infants (P21, P25, P27) lack of data prevented us to unquestionably assign them to group 2 and 3. Hence, they were categorized as “unclassified”.

False positives accounted for 41.3% of recalled newborns and 0.012% of the total number of newborns born in the study period in the present study (Fig. 2). Test performance of NBS for CUD has varied significantly across regions. Positive predictive values ranged from 1.63% in Bavaria [10] and 1.93% in China [20] to 2.4% in New Zealand [12] and 4.7% in California [11]. High percentage of false positive is a major issue of NBS for CUD. Possible determinants include blood samples collected too early after birth [11], early gestational age, low birth weight, exclusive breastfeeding, caesarean delivery. Two infants included in the false positive group (P45 e P46) were preterm newborns in the present study (Table 4). Gestational age is known to influence C0 levels in newborns. Particularly, a significant decrease in free carnitine levels was found in the first weeks of life of infants born < 37 weeks [38, 39]. This observation would argue in favor of the use of carnitine-enriched parenteral nutrition in preterm infants. However, little is known about the clinical efficacy of this prophylactic approach, and further studies are required [40]. Among false positive newborns, three had a low birth weight (P44,P45,P46) in the present study (Table 4). Birth weight can also affect C0 levels in newborns. Low C0 has been found in infants with birth weight < 1800 g [14, 15]. Future studies evaluating the benefit of using specific cutoff values for preterm/low birth weight infants are warranted. 10/19 (53%) of false positive newborns, were born by caesarean section in the present study (Table 4). Caesarean delivery is an additional possible concurring factor potentially inducing temporary upregulation of FAO resulting in free carnitine depletion in the newborn [41]. Although the role of each of these additional factors could not be dissected in the present study, we speculate that low C0 was the result of the combination of the above-mentioned contributors. Future studies investigating the effect of these determinants as well as additional perinatal stresses (e.g. respiratory distress, hypoxia, infection) on C0 concentrations in newborns are warranted.

Overall, data from the present study showed that NBS can identify not only (i) children with CUD but also (ii) those whose mothers have carnitine deficiency and (iii) heterozygous carriers, for whom monitoring may (not) be required. Remarkably, a high percentage of false positives was detected. Even though the rate of false positives was in line with previous studies, our results highlight the need to increase the NBS specificity to ensure its sustainability [22–24, 42–49]. Recently, the ratio of urine C0/plasma C0 has been proposed as a helpful biomarker to discriminate between positive and false positive newborn referrals [42]. Furthermore, regular revision of diagnostic cutoffs [47] as well as the systematic use of (first-/second-tier) genotyping combined with biochemical phenotyping on newborns and mothers [48, 49], ought to be adopted in each NBS program to decrease the false positive rate [28, 48–55].

## Conclusions

NBS for CUD can detect affected children, infants born to mothers with carnitine deficiency and heterozygous individuals. However, it is still burdened by a high false positive rate. In this study we presented the outcomes of such program related to the identification of (un)affected newborns/mothers and its limitations due to low specificity. The concurrent evaluation of C0 and C_tot_ values can increase the NBS efficiency. In all cases of newborns recalled for low C0, maternal acylcarnitine profile should be ordered. We recommend *SLC22A5* molecular testing in all newborns recalled for low C0 displaying C_tot_ values ≤ 19 µmol/L. Molecular testing can be considered in other cases. Possible additional strategies to minimize false positives and avoid unnecessary recalls are linked to the regular revision of diagnostic cutoffs, assessment of fractional excretion of carnitine and implementation of new analytical strategies in the development of second-tier tests.

## Supplementary Information

Below is the link to the electronic supplementary material.

Supplementary Material 1

## Data Availability

The data generated in this study are available from the corresponding author upon reasonable request.

## References

[1] Juraszek B, Nałęcz KA. SLC22A5 (OCTN2) carnitine Transporter-Indispensable for cell Metabolism, a Jekyll and Hyde of human cancer. Molecules. 2019;25(1):14. 10.3390/molecules25010014. 31861504 10.3390/molecules25010014 PMC6982704

[2] Almannai M, Alfadhel M, El-Hattab AW. Carnitine inborn errors of metabolism. Molecules. 2019;24(18):3251. 10.3390/molecules24183251. 31500110 10.3390/molecules24183251 PMC6766900

[3] Cemeroglu AP, Kocabaş CN, Coşkun T, Gürgey A. Low serum carnitine concentrations in healthy children with iron deficiency anemia. Pediatr Hematol Oncol. 2001;18(8):491–5. 10.1080/088800101753328457. 11764098 10.1080/088800101753328457

[4] Rasmussen J, Nielsen OW, Lund AM, Køber L, Djurhuus H. Primary carnitine deficiency and pivalic acid exposure causing encephalopathy and fatal cardiac events. J Inherit Metab Dis. 2013;36(1):35–41. 10.1007/s10545-012-9488-8. 22566287 10.1007/s10545-012-9488-8

[5] Spiekerkoetter U, Huener G, Baykal T, Demirkol M, Duran M, Wanders R, Nezu J, Mayatepek E. Silent and symptomatic primary carnitine deficiency within the same family due to identical mutations in the organic cation/carnitine transporter OCTN2. J Inherit Metab Dis. 2003;26:613–5. 10.1023/A:1025968502527. 14605509 10.1023/a:1025968502527

[6] Vijay S, Patterson A, Olpin S, Henderson MJ, Clark S, Day C, Savill G, Walter JH. Carnitine transporter defect: diagnosis in asymptomatic adult women following analysis of acylcarnitines in their newborn infants. J Inherit Metab Dis. 2006;29(5):627–30. 10.1007/s10545-006-0376-y. 16865412 10.1007/s10545-006-0376-y

[7] El-Hattab AW, et al. Systemic primary carnitine deficiency. In: Adam MP, Everman DB, Mirzaa GM, editors. GeneReviews^®^ [Internet]. Seattle (WA): University of Washington, Seattle; 1993–2023. 22420015

[8] Berry SA. – 53, x. Newborn Screen Clin Perinatol. 2015;42(2):441–. 10.1016/j.clp.2015.03.002. 26042913 10.1016/j.clp.2015.03.002

[9] Magnuolo A, Rodella G, Dianin A, Nurti R, Monge I, Rigotti E, Cantalupo G, Salviati L, Tucci S, Pellegrini F, Molinaro G, Lupi F, Tonin P, Pasini A, Campostrini N, Ion Popa F, Teofoli F, Vincenzi M, Camiloti M, Piacentini G, Bordugo A. Diagnosis, genetic characterization and clinical follow-up of mitochondrial fatty acid oxidation disorders in the new era of expanded newborn screening: A single centre experience. Mol Genet Metab Rep. 2020;24:100632. 10.1016/j.ymgmr.2020.100632. 32793418 10.1016/j.ymgmr.2020.100632 PMC7414009

[10] Schiergens KA, Weiss KJ, Roschinger W, Lotz-Havla AS, Schmitt J, Dalla Pozza R, Ulrich S, Odenwald B, Kreuder J, Maier EM. Newborn screening for carnitine transporter defect in Bavaria and the long-term follow-up of the identified newborns and mothers: assessing the benefit and possible harm based on 19 ½ years of experience. Mol Genet Metabolism Rep. 2020;24:100632. 10.1016/j.ymgmr.2021.100776 PMC8214137 34178604

[11] Gallant NM, Leydiker K, Wilnai Y, Lee C, Lorey F, Feuchtbaum L, Tang H, Carter J, Enns GM, Packman S, Lin HJ, Wilcox ER, Cederbaum SD, Abdenur JE. Biochemical characteristics of newborns with carnitine transporter defect identified by newborn screening in California. Mol Genet Metab. 2017;122(3):76–84. 10.1016/j.ymgme.2017.06.015. 28711408 10.1016/j.ymgme.2017.06.015

[12] Wilson C, Knoll D, de Hora M, Kyle C, Glamuzina E, Webster D. The decision to discontinue screening for carnitine uptake disorder in new Zealand. J Inherit Metab Dis. 2019;42(1):86–92. 10.1002/jimd.12030. 30740730 10.1002/jimd.12030

[13] Rossi A, Hoogeveen IJ, Lubout CMA, de Boer F, Fokkert-Wilts MJ, Rodenburg IL, van Dam E, Grünert SC, Martinelli D, Scarpa M, CONNECT MetabERN Collaboration Group, Dekker H, te Boekhorst ST, van Spronsen FJ, Derks TGJ. A generic emergency protocol for patients with inborn errors of metabolism causing fasting intolerance: A retrospective, single-center study and the generation of www.emergencyprotocol.net. Inherit Metab Dis. 2021;44:1124–35. 10.1002/jimd.12386 PMC8518720 33844307

[14] Baronio F, Righi B, Righetti F, Bettocchi I, Ortolano R, Faldella G, Rondelli R, Pession A, Cassio A. Carnitine longitudinal pattern in preterm infants < 1800 g body weight: a case–control study. Pediatr Res. 2019;86:646–50. 31291643 10.1038/s41390-019-0497-2

[15] Bene J, et al. Differences in Circulating carnitine status of preterm infants fed fortified human milk or preterm infant formula. J Pediatr Gastroenterol Nutr. 2013;57:673–6. 23783025 10.1097/MPG.0b013e31829fad06

[16] Scolamiero E, Cozzolino C, Albano L, Ansalone A, Caterino M, Corbo G, di Girolamo MG, Di Stefano C, Durante A, Franzese G, Franzese I, Gallo G, Giliberti P, Ingenito L, Ippolito G, Malamisura B, Mazzeo P, Norma A, Ombrone D, Parenti GC, Pellecchia S, Pecce R, Pierucci I, Romanelli R, Rossi A, Siano M, Stoduto T, Villa G, Andria G, Salvatore F, Frisso G, Ruoppolo M. Targeted metabolomics in the expanded newborn screening for inborn errors of metabolism. Mol Biosyst. 2015;11(6):1525–35. 10.1039/c4mb00729h. 25689098 10.1039/c4mb00729h

[17] Lombardo B, D’Argenio V, Monda E, Vitale A, Caiazza M, Sacchetti L, Pastore L, Limongelli G, Frisso G, Mazzaccara C. Genetic analysis resolves differential diagnosis of a Familial syndromic dilated cardiomyopathy: A new case of Alström syndrome. Mol Genet Genomic Med. 2020;8(7):e1260. 10.1002/mgg3.1260. 32396277 10.1002/mgg3.1260 PMC7336746

[18] Richards S, Aziz N, Bale S, Bick D, Das S, Gastier-Foster J, Grody WW, Hegde M, Lyon E, Spector E, Voelkerding K, Rehm HL, ACMG Laboratory Quality Assurance Committee. Standards and guidelines for the interpretation of sequence variants: a joint consensus recommendation of the American college of medical genetics and genomics and the association for molecular pathology. Genet Med. 2015;17(5):405–24. 25741868 10.1038/gim.2015.30 PMC4544753

[19] Pejaver V, Byrne AB, Feng BJ, Pagel KA, Mooney SD, Karchin R, O’Donnell-Luria A, Harrison SM, Tavtigian SV, Greenblatt MS, Biesecker LG, Radivojac P, Brenner SE, ClinGen Sequence Variant Interpretation Working Group. Calibration of computational tools for missense variant pathogenicity classification and ClinGen recommendations for PP3/BP4 criteria. Am J Hum Genet. 2022;109(12):2163–77. 10.1016/j.ajhg.2022.10.013. 36413997 10.1016/j.ajhg.2022.10.013 PMC9748256

[20] Lin Y, Xu H, Zhou D, Hua Z, Zhang C, Zhou W, Zhang L, Huang T, Li W. Screening 3.4 million newborns for primary carnitine deficiency in Zhejiang Province, China. Clin Chim Acta. 2020;507:199–204. 32371215 10.1016/j.cca.2020.04.039

[21] Lin W, Wang K, Zheng Z, Chen Y, Fu C, Lin Y, Chen D. Newborn screening for primary carnitine deficiency in Quanzhou, China. Clin Chim Acta. 2021;512:166–71. 33181153 10.1016/j.cca.2020.11.005

[22] Crefcoeur LL, Visser G, Ferdinandusse S, Wijburg FA, Langeveld M, Sjouke B. Clinical characteristics of primary carnitine deficiency: A structured review using a case-by-case approach. J Inherit Metab Dis. 2022;45:386–405. 34997761 10.1002/jimd.12475 PMC9305179

[23] Liammongkolkul S, Boonyawat B, Vijiarnsorn C, Tim-Aroon T, Wasant P, Vatanavicharn N. Phenotypic and molecular features of Thai patients with primary carnitine deficiency. Pediatr Int. 2023;65(1):e15404. 36321377 10.1111/ped.15404

[24] Lin W, Wang K, Zheng Z, Chen Y, Fu C, Lin Y, Chen D, Rasmussen J, Lund AM, Risom L, Wibrand F, Gislason H, Nielsen OW, Køber L, Duno M. Residual OCTN2 transporter activity, carnitine levels and symptoms correlate in patients with primary carnitine deficiency. Mol Genet Metab. 2014;241–8. 10.1016/j.ymgmr.2014.04.008 PMC5121291 27896095

[25] Ruoppolo M, et al. Expanded newborn screening in Italy using tandem mass spectrometry: two years of National experience. Int J Neonatal Screen. 2022;8:47. 10.3390/ijns8030047. 35997437 10.3390/ijns8030047 PMC9397032

[26] Frigeni M, Balakrishnan B, Yin X, Calderon FRO, Mao R, Pasquali M, Longo N. Functional and molecular studies in primary carnitine deficiency. Hum Mutat. 2017;38(12):1684–99. 10.1002/humu.23315. 28841266 10.1002/humu.23315 PMC5665702

[27] Koleske ML, McInnes G, Brown JEH, Thomas N, Hutchinson K, Chin MY, Koehl A, Arkin MR, Schlessinger A, Gallagher RC, Song YS, Altman RB, Giacomini KM. Functional genomics of OCTN2 variants informs protein-specific variant effect predictor for carnitine transporter deficiency. Proc Natl Acad Sci U S A. 2022;119(46):e2210247119. 10.1073/pnas.2210247119. 36343260 10.1073/pnas.2210247119 PMC9674959

[28] Crefcoeur L, Ferdinandusse S, van der Crabben SN, Dekkers E, Fuchs SA, Huidekoper H, Janssen M, Langendonk JL, Maase R, de Sain M, Rubio E, van Spronsen FJ, Vaz FM, Verschoof R, de Vries M, Wijburg F, Visser G, Langeveld M. Newborn screening for primary carnitine deficiency: who will benefit? A retrospective cohort study. J Med Genet. 2023;0:1–9. 10.1136/jmg-2023-109206. 10.1136/jmg-2023-109206 PMC10715524 37487700

[29] Hertz CL, Christiansen SL, Larsen MK, Dahl M, Ferrero-Miliani L, Weeke PE, Pedersen O, Hansen T, Grarup N, Ottesen GL, Frank-Hansen R, Banner J, Morling N. Genetic investigations of sudden unexpected deaths in infancy using next-generation sequencing of 100 genes associated with cardiac diseases. Eur J Hum Genet. 2016;24(6):817–22. 10.1038/ejhg.2015.198. 26350513 10.1038/ejhg.2015.198 PMC4867441

[30] Neubauer J, Lecca MR, Russo G, Bartsch C, Medeiros-Domingo A, Berger W, Haas C. Post-mortem whole-exome analysis in a large sudden infant death syndrome cohort with a focus on cardiovascular and metabolic genetic diseases. Eur J Hum Genet. 2017;25(4):404–9. 10.1038/ejhg.2016.199. 28074886 10.1038/ejhg.2016.199 PMC5386419

[31] Barbosa-Gouveia S, Vázquez-Mosquera ME, González-Vioque E, Álvarez JV, Chans R, Laranjeira F, Martins E, Ferreira AC, Avila-Alvarez A, Couce ML. Utility of gene panels for the diagnosis of inborn errors of metabolism in a metabolic reference center. Genes (Basel). 2021;12(8):1262. 10.3390/genes12081262. 34440436 10.3390/genes12081262 PMC8391361

[32] Jakoby M 4th, Jaju A, Marsh A, Wilber A. Maternal primary carnitine deficiency and a novel solute carrier family 22 member 5 (SLC22A5) mutation. J Investig Med High Impact Case Rep. 2021 Jan-Dec;9:23247096211019543. 10.1177/23247096211019543. 10.1177/23247096211019543 PMC8155745 34032155

[33] Li F-Y, El-Hattab AW, Bawle EV, Boles RG, Schmitt ES, Scaglia F, Wong LJ. Molecular spectrum of SLC22A5 (OCTN2) gene mutations detected in 143 subjects evaluated for systemic carnitine deficiency. Hum Mutat. 2010;31(8):E1632–51. 10.1002/humu.21311. 20574985 10.1002/humu.21311

[34] Ji X, Ge Y, Ni Q, Xu S, Xiong Z, Yang L, Hu L, Cao Y, Lu Y, Wei Q, Kang W, Zhuang D, Zhou W, Dong X. Primary carnitine deficiency: Estimation of prevalence in Chinese population and insights into newborn screening. Front Genet. 2023;14:1304458. 10.3389/fgene.2023.1304458. 38125748 10.3389/fgene.2023.1304458 PMC10730660

[35] Verbeeten KC, Lam-honwah AM, Bulman D, Faghfoury H, Chakraborty P, Tein I, Geraghty MT. Carnitine uptake defect due to a 5’UTR mutation in a pedigree with false positives and false negatives on newborn screening. Mol Genet Metab. 2020;129(3):213–8. 31864849 10.1016/j.ymgme.2019.12.006

[36] Crombez EA, Cederbaum SD, Spector ES, Chan E, Salazar D, Neidich J, Goodman S. Maternal glutaric Acidemia, type I identified by newborn screening. Mol Genet Metab. 2008;94(1):132–4. 18304851 10.1016/j.ymgme.2008.01.005 PMC2474772

[37] Sarafoglou K, Tridgell AHC, Bentler K, Redlinger-Grosse K, Berry SA, Schimmenti LA. Cardiac conduction improvement in two heterozygotes for primary carnitine deficiency on l-carnitine supplementation. Clin Genet. 2010;78:191–4. 20095986 10.1111/j.1399-0004.2009.01368.x

[38] Mandour I, et al. Amino acid and acylcarnitine profiles in premature neonates: a pilot study. Indian J Pediatr. 2013;80(9):736–44. 10.1007/s12098-013-0980-4. 23404695 10.1007/s12098-013-0980-4

[39] Lefèvre CR, Labarthe F, Philippe G, Harambat J, Parvy P, Dore L, Tordjman M, Chabrol B, Gagnadoux MF. Liver transplant for primary carnitine deficiency. J Hepatol. 2004;40(4):657–9.

[40] Salguero Olid A, Blanco Sánchez G, Alonso Ojembarrena A. A systematic review about prophylactic L-carnitine administration in parenteral nutrition of extremely preterm infants. Farm Hosp. 2018;42(4):168–73. 29959842 10.7399/fh.10976

[41] Marchioro L, Shokry E, Geraghty AA, O’Brien EC, Uhl O, Koletzko B, McAulife FM. Caesarean section, but not induction of labour, is associated with major changes in cord blood metabolome. Sci Rep. 2019;9:17562. 31772287 10.1038/s41598-019-53810-1 PMC6879512

[42] Crefcoeur LL, Heiner-Fokkema MR, Maase RE, Visser G, de Sain-van der Velden MG. Assessment of carnitine excretion and its ratio to plasma free carnitine as a biomarker for primary carnitine deficiency in newborns. JIMD Rep. 2023;64:57–64. 36636597 10.1002/jmd2.12334 PMC9830017

[43] Lin Y, Lin B, Chen Y, Zheng Z, Fu Q, Lin W, Zhang W. Biochemical and genetic characteristics of patients with primary carnitine deficiency identified through newborn screening. Orphanet J Rare Dis. 2021;16:503. 34863234 10.1186/s13023-021-02126-3 PMC8642906

[44] Adhikari AN, Gallagher RC, Wang Y, Currier RJ, Amatuni G, Bassaganyas L, Chen F, Kundu K, Kvale M, Mooney SD, Nussbaum RL, Randi SS, Sanford J, Shieh JT, Srinivasan R, Sunderam U, Vaka D, Zou Y, Koenig BA, Kwok PY, Risch N, Puck JM, Brenner SE. The role of exome sequencing in newborn screening for inborn errors of metabolism. Nat Med. 2020;26(9):1392–7. 10.1038/s41591-020-0966-5. 32778825 10.1038/s41591-020-0966-5 PMC8800147

[45] Kingsmore SF, Smith LD, Kunard CM, Willis MJ, Wolen AR, Defay T. A genome sequencing system for universal newborn screening, diagnosis, and precision medicine for severe genetic diseases. AJHG. 2022;111(2):135–45. 10.1016/j.ajhg.2022.08.003. 10.1016/j.ajhg.2022.08.003 PMC9502059 36007526

[46] Sun Y, Wang Y, Jiang T. Clinical features and genotyping of patients with primary carnitine deficiency identified by newborn screening. J Pediatr Endocrinol Metab. 2017;30(7):809–15. 10.1515/jpem-2017-0002 28753539

[47] Malvagia S, Forni G, Ombrone D, la Marca G. Development of strategies to decrease false positive results in newborn screening. Int J Neonatal Screen. 2020;6(1):45. 33147868 10.3390/ijns6040084 PMC7712114

[48] Wang LY, Chen NI, Chen PW, Chiang SC, Hwu WL, Lee NC, Chien YH. Newborn screening for Citrin deficiency and carnitine uptake defect using second-tier molecular tests. BMC Med Genet. 2013;14:24. 10.1186/1471-2350-14-24. 23394329 10.1186/1471-2350-14-24 PMC3575349

[49] van den Heuvel LM, Kater-Kuipers A, van Dijk T, Crefcoeur L, Visser G, Langeveld M, Henneman L. A qualitative study on the perspectives of mothers who had been diagnosed with primary carnitine deficiency through newborn screening of their child. Orphanet J Rare Dis. 2023;18(1):134. 10.1186/s13023-023-02735-0. 37268964 10.1186/s13023-023-02735-0 PMC10236393

[50] Zhou J, Li G, Zeng Y, Qiu X, Zhao P, Huang T, Wang X, Luo J, Lin N, Xu L. Screening primary carnitine deficiency in 10 million Chinese newborns: a systematic review and meta-analysis. Orphanet J Rare Dis. 2024;19(1):248. 10.1186/s13023-024-03267-x. 38961493 10.1186/s13023-024-03267-x PMC11220949

[51] Lin Y, Lin C, Zheng Z, Huang C, Peng W. Newborn screening for primary carnitine deficiency using a second-tier genetic test. J Pediatr Endocrinol Metab. 2024;37(2):163–9. 10.1515/jpem-2023-0513. 38158618 10.1515/jpem-2023-0513

[52] Lin Y, Zheng Z, Lin W, Peng W. Incorporating Next-Generation sequencing as a Second-Tier test for primary carnitine deficiency. Mol Genet Genomic Med. 2024;12(9):e70003. 10.1002/mgg3.70003. 39248612 10.1002/mgg3.70003 PMC11382357

[53] Abrahamsen RK, Lund AM, Rasmussen J. Patients with primary carnitine deficiency treated with L-carnitine are alive and doing well: A 10-year follow-up in the Faroe Islands. JIMD Rep. 2023;64(6):453–9. 10.1002/jmd2.12383. 37927485 10.1002/jmd2.12383 PMC10623095

[54] Hu H, Ma Q, Wang Y, Song W, Xu H. Newborn screening of primary carnitine deficiency: clinical and molecular genetic characteristics. Ital J Pediatr. 2025;51(1):70. 10.1186/s13052-025-01911-1. 40069787 10.1186/s13052-025-01911-1 PMC11900477

[55] Lin Y, Zhang W, Huang C, Lin C, Lin W, Peng W, Fu Q, Chen D. Increased detection of primary carnitine deficiency through second-tier newborn genetic screening. Orphanet J Rare Dis. 2021;16:149. 33757571 10.1186/s13023-021-01785-6 PMC7988980

