# Supplementary material for: Benefits and pitfalls in newborn screening for carnitine uptake deficiency: a 4-year single-center experience
Source: Orphanet J Rare Dis. 2025 Nov 28;21:100. doi: 10.1186/s13023-025-04133-0 (PMC13003709; doi:10.1186/s13023-025-04133-0)
Supplement: Supplementary file 1 — Supplementary Material 1 [file 13023_2025_4133_MOESM1_ESM.docx]

YES

YES

YES

SPOT BLOOD SAMPLING between the 48^th^ and the 72^th^ hour of newborn’s life

DELIVERY OF THE SAMPLE TO THECOURIER

WAS SPOT BLOOD DONE CORRECTLY?

REQUEST FOR NEW SAMPLE AT THE BIRTHCENTER

SCREENING REPORTare sent to newborn centres for negative and positive cases

SAMPLE ACCEPTANCE

NEWBORN RECALL AND NEW SPOT BLOOD COLLECTION

SPOT ANALYSIS

POSITIVITY TO ANALYTES?

EMERGENCY SITUATION?

DIRECT NEWBORN RECALL FROM THE CLINICAL CENTER

Ì

NO

NO

NO

Diagnostic tests of 2nd level. Mother is asked to test her acylcarnitines.

POSITIVITY TO ANALYTES?

NO

MOLECULAR ANALYSIS (on peripheral blood sample). L-CARNITINE supplementation

YES

**Supplemental Figure 1.Regional Operative Procedure for newborns suspected with Carnitine uptake deficiency at newborn screening**

YES

YES

YES
